# Radiological Investigation of Acute Renal Colic: A Two-Cycle Clinical Audit

**DOI:** 10.1055/s-0045-1814737

**Published:** 2025-12-31

**Authors:** Ibrahim Abuelbeh, Mohammed Nawaiseh, Hussam Nawaiseh, Yahia Metwally, Qais Nawaiseh, Sa'id Dabah Aljamal, Anas Hattab, Yousef Alwan, Thomas Brophy, Ian Pearce, Vaibhav Modgil

**Affiliations:** 1 Department of Urology, Manchester University NHS Foundation Trust, Manchester, United Kingdom; 2 Department of Ophthalmology, Jordanian Royal Medical Services, Amman, Jordan; 3 Department of Gastroenterology, Mid Cheshire NHS Foundation Trust, Crewe, United Kingdom; 4 School of Medicine, The University of Jordan, Amman, Jordan; 5 Department of Radiology, Manchester University NHS Foundation Trust, Manchester, United Kingdom

**Keywords:** renal colic, CT KUB, clinical audit, imaging guidelines, urolithiasis, urinary tract stones

## Abstract

**Background:**

Renal colic is a common urological emergency that requires a noncontrast computed tomography of the kidneys, ureters, and bladder (CT KUB) as the investigation of choice. This two-cycle audit was conducted at Manchester Royal Infirmary; a tertiary hospital in the United Kingdom. It evaluates the appropriateness of CT KUB imaging for acute renal colic, focusing on scan timing, diagnostic yield, and request adequacy in line with national guidelines.

**Methods:**

We performed a retrospective review of patients referred from the accident and emergency department for suspected renal colic. The first cycle was conducted in September to October 2022 and the second in August 2023. The intervention included disseminating the findings and designing an educational program. Statistical analysis was performed using SPSS version 30.0. Continuous variables were compared using independent *t* -tests, and categorical variables using chi-square tests. A *p* -value of < 0.05 was considered statistically significant.

**Results:**

A total of 153 patients were included (first cycle: 77; second cycle: 76). Following the intervention, the mean time from presentation to CT KUB report decreased significantly from 14.7 ± 9.5 to 8.2 ± 5.1 hours ( *p* < 0.001). The proportion of scans reported within 24 hours also increased from 92.2 to 100% ( *p* = 0.007). Documentation of a sufficient history improved from 76.6 to 89.5% ( *p* = 0.034). Additionally, reports of nonspecific abdominal pain decreased from 9.1 to 0% ( *p* = 0.007).

**Conclusion:**

Targeted educational interventions improved CT KUB timeliness, request quality, and adherence to national guidelines. These results confirm the value of structured clinical pathways and cross-departmental collaboration in optimizing the investigation of suspected renal colic.

## Introduction

Renal colic is characterized by the acute onset of intense flank pain resulting from obstruction within the urinary tract, mainly due to urolithiasis. The pain is frequently described as radiating from the flank to the groin or “loin to groin” and may be accompanied by symptoms such as nausea, vomiting, and hematuria. As the stone progresses along the ureter, additional symptoms may arise. These can include urinary urgency, dysuria, and systemic features such as fever and tachycardia, that may suggest superimposed infection. 1 2

Renal colic is a prevalent condition, with an estimated lifetime incidence of up to 12% in men and 6% in women. Without definitive treatment or risk factor modification, recurrence rates can reach 50%. In recent decades, the global burden of kidney stone disease has risen, likely due to dietary changes, increasing obesity, and climate factors. Timely and accurate diagnosis is crucial. Delays in diagnosis or management can lead to serious complications such as urosepsis, renal impairment, or even death. In cases of obstructive uropathy with infection, delaying decompression increases the risk of death by approximately 29%. 3 4 5 6

National guidelines from both the British Association of Urological Surgeons (BAUS) and the National Institute for Health and Care Excellence (NICE) recommend imaging within 24 hours for patients with suspected renal colic, ideally using a low-dose, noncontrast computed tomography of the kidneys, ureters, and bladder (CT KUB). CT KUB offers superior sensitivity and specificity for detecting urolithiasis compared with plain abdominal radiographs, ultrasound, and magnetic resonance imaging. However, despite being termed “low-dose,” this modality still involves appreciable radiation exposure. Consequently, the Royal College of Radiologists (RCR) emphasizes auditing CT KUB usage to ensure each scan is appropriately justified and in line with best-practice guidelines. 7 8 9

This study presents a two-cycle clinical audit conducted at Manchester Royal Infirmary, a tertiary hospital in England to evaluate compliance with national imaging guidelines for investigating suspected renal colic. By identifying deviations from protocol and implementing targeted interventions, the project aimed to enhance diagnostic accuracy, minimize unnecessary radiation exposure, and improve overall patient care. A reaudit cycle was performed to assess the effectiveness of these changes and complete the audit loop.

## Methods

Institutional approval was obtained from the Clinical Audit and Effectiveness Department to ensure compliance with ethical and governance standards. This project involved a detailed retrospective review of CT KUB imaging performed at a tertiary hospital for the evaluation of patients with suspected renal colic. All data were anonymized to safeguard patients' confidentiality and avoid including any identifiable information.

The audit was conducted in two cycles. The first cycle reviewed scans performed from September to October 2022, while the reaudit covered August 2023. In each cycle, data collection continued until at least 75 eligible patients were included. Patients under the age of 18 and pregnant individuals were excluded from the study, as CT KUB is generally avoided in these groups due to the increased risk of radiation exposure. Children are more sensitive to radiation and have a longer lifetime risk of radiation-induced malignancy. Similarly, in pregnant patients, fetal radiation exposure carries teratogenic and oncogenic risks. Consequently, current guidelines recommend ultrasound as the preferred first-line imaging in these populations. 8 10 11

Additionally, patients presenting with renal colic who had a prior CT KUB within the preceding 3 months were excluded. For these individuals, both RCR and BAUS recommend repeat imaging with either ultrasound or plain abdominal X-ray to limit cumulative radiation exposure. Since CT KUB is not routinely indicated in such cases, patients were excluded from the audit. 7 9 12

During the first cycle, 77 CT KUB cases were reviewed, following the exclusion of patients under 18 and pregnant patients, a total of 43 males, 33 females, and one nonbinary. Collected data included patients' age and gender, information provided in the imaging request, referral source and date, and radiology findings. Also, data of whether imaging occurred within 24 hours of presentation, along with radiology report time were collected. The results were assessed against national standards from NICE, BAUS, and RCR. According to these guidelines, all eligible patients should undergo CT KUB within 24 hours of presentation. Additionally, 44 to 64% of scans should detect renal calculi, and 6 to 18% should reveal alternative diagnoses. All scans were requested from the accident and emergency (A&E) department as per the Emergency Department Atraumatic Loin Pain Pathway. This pathway was introduced in the hospital in 2015 and allows A&E clinicians to directly order CT KUB without prior urology consultation. Primary care physicians also utilize this pathway by referring suspected renal colic patients to A&E for investigation. 7 8 9

Following the first cycle, findings were shared with the urology, emergency medicine, and radiology teams. Additionally, a presentation was made during the hospital departmental governance meeting. Recommendations were proposed and implemented to enhance the quality of care, with a primary focus on educational interventions. Refresher sessions and posters were provided for both medical and allied health staff, along with clear signposting to relevant clinical guidelines and protocols. Particular attention was given to highlighting key clinical indicators suggestive of renal colic, identified through comprehensive clinical assessments, to support a more effective vetting process. This information was also disseminated to primary care providers via designated representatives.

A second audit was conducted to evaluate the impact of the implemented interventions. A sample of 76 patients, comprising 40 males and 36 females, was collected using the same inclusion and exclusion criteria as the first cycle. Data were gathered using the same approach as in the initial cycle. Two consultant radiologists supervised both cycles to ensure accuracy, consistency, and integrity.

Descriptive statistics were used to summarize the data. Continuous variables, such as age and overall timing from presentation to writing the report, were reported as means with standard deviations. Categorical variables were summarized using frequencies and percentages. To compare variables between the first and second audit cycles, independent *t* -tests were applied for continuous variables, while the chi-square test was used for categorical variables. A two-tailed *p* -value of < 0.05 was considered statistically significant. Statistical analyses were conducted using Statistical Package for the Social Sciences [SPSS] version 30.0.

## Results

A total of 153 patients were included in the study, with 77 (50.3%) in the first cycle and 76 (49.7%) in the second cycle. The mean age of patients was similar between the two cycles (39.6 ± 14.2 vs. 39.0 ± 13.3 years; *p* = 0.777). There was no significant difference in gender distribution ( *p* = 0.540), with males comprising 55.8% in cycle 1 and 52.6% in cycle 2. One patient (1.3%) in the first cycle identified as nonbinary. A significant improvement was observed in the overall timing from presentation to CT KUB report, decreasing from a mean of 14.7 ± 9.5 hours in the first cycle to 8.2 ± 5.1 hours in the second cycle ( *p* < 0.001). The proportion of patients whose scans were reported within 24 hours also increased significantly from 92.2 to 100% ( *p* = 0.007). Patient demographics and key outcome measures before and after the intervention are summarized in Table 1 .

Regarding clinical presentation, the documentation of sufficient history improved from 76.6% in the first cycle to 89.5% in the second cycle ( *p* = 0.034). Loin pain was more frequently reported in the second cycle (98.7%) compared with the first (90.9%) ( *p* = 0.031), while the incidence of nonspecific abdominal pain decreased significantly from 9.1 to 0% ( *p* = 0.007). The prevalence of hematuria remained comparable between cycles ( *p* = 0.777).

There were no significant differences between cycles in the detection of renal stones (41.6% vs. 48.7%; *p* = 0.376) or alternative diagnoses (10.3% vs. 17.1%; *p* = 0.674). However, a significantly lower proportion of patients had undergone previous CT KUB imaging in the second cycle (9.2%) compared with the first (22.1%) ( *p* = 0.029).

## Discussion

In this clinical audit we aimed to improve clinical practice in line with evidence-based standards from NICE, BAUS, and RCR. Specifically, we evaluated compliance with three key standards for CT KUB requests in acute renal colic. First, whether CT KUB was performed within 24 hours of presentation. Second, whether the diagnostic yield of CT KUB met national benchmarks, defined as a positive stone detection rate of 44 to 64% and an alternative diagnosis rate of 6 to 18%. Third, whether imaging requests included a sufficient clinical history. Through this two-cycle audit and a targeted educational intervention, we sought to enhance both the appropriateness and timeliness of imaging for suspected renal colic. 7 8 9

Epidemiological studies report a higher incidence of renal colic in males, particularly those aged 40 to 50 years. In our audit, patient demographics were consistent with these trends. Across both cycles, the average patient age remained stable: 39.6 years in the first cycle and 39.0 years in the second, indicating a predominance of cases in the middle-aged adult population. A slight male predominance was also observed, with males comprising 55.8% of patients in the first cycle and 52.6% in the second. This distribution aligns with the known epidemiology of urolithiasis and supports the generalizability of our findings to standard adult populations presenting with suspected renal colic. 13 14 15

In the first cycle of our audit, several deviations from national imaging standards were identified. Although 92.2% of patients received CT KUB within the recommended 24-hour window, the diagnostic yield for renal stones was 41.6%, slightly below the lower threshold of the expected benchmark (44–64%). Additionally, 9.1% of patients in this cycle were scanned despite presenting with nonspecific abdominal pain, suggesting that imaging was sometimes used to investigate a broad range of differential diagnoses rather than to confirm clinically suspected renal colic. This likely reflects inconsistencies in clinical documentation and a lack of targeted symptom reporting in CT requests. A lack of awareness of the up-to-date guidelines, combined with reliance on imaging to diagnose nonspecific abdominal pain rather than confirming suspected urolithiasis in renal colic patients might also explain the finding. Additionally, the vetting process was not as robust as it should have been, as many patients were investigated with the inappropriate imaging modality, in an attempt to explore various possible causes. Inadequate clinical information can hinder appropriate vetting, potentially leading to unnecessary imaging and suboptimal use of radiological resources. These findings underscore the need to reinforce education on proper referral practices and emphasize the importance of providing clear, focused clinical histories that align with guideline-based imaging criteria.

Our first-cycle findings align with those of other studies evaluating CT KUB use for investigating renal colic. Two U.K.-based studies reported CT KUB urolithiasis positive detection rates of 47.5 and 41.2%, respectively, with alternative diagnoses identified in 10 and 9.8% of cases. In comparison, our first audit cycle had a stone detection rate of 41.6% and an alternative diagnosis rate of 10.3%, which aligns with these figures. Likewise, those studies recommended further improvements in CT KUB request practices. 14 16

In response to the first-cycle findings, we implemented a refresher educational program targeting key clinical departments involved in the management of renal colic. Refresher sessions were delivered for staff in the urology, radiology, and emergency medicine departments to reinforce best practices for imaging indications, documentation, and guideline adherence. These sessions focused on improving the clarity and clinical relevance of CT KUB requests, with particular emphasis on identifying hallmark features of renal colic, such as acute flank pain radiating to the groin, hematuria, and associated nausea, vomiting, or urinary symptoms. Additionally, reference posters were distributed in the A&E department for quick and easy access. To support these initiatives, the audit findings were formally presented at urology, emergency medicine, and radiology departmental meetings, facilitating wider discussion and encouraging multidisciplinary engagement. Staff were also reminded of the exclusion criteria and the importance of avoiding unnecessary imaging, especially in patients who have been recently imaged or in whom radiation exposure carries a higher risk. This collaborative approach aimed to strengthen clinical decision-making, promote more accurate vetting of imaging requests, and align departmental practice with national guidelines.

As part of our intervention, we reinforced the importance of adhering to national guidelines issued by the NICE, RCR, and BAUS, particularly the recommendation that patients with suspected renal colic should undergo imaging within 24 hours of presentation. Emphasis was placed on how timely and appropriate investigation can lead to earlier diagnosis, reduce the risk of complications, and improve overall patient outcomes. The impact of these measures was evident in our second audit cycle, which demonstrated a significant improvement in compliance with the 24-hour imaging standard and an increased CT KUB positive detection rate of urinary calculi. These findings highlight the effectiveness of our educational efforts in promoting evidence-based practice and improving the quality of care delivered.

Compared with the audit conducted at Newham University Hospital (NUH), our project shares a similar structure and objectives, aiming to improve compliance with national guidelines for the radiological investigation of suspected renal colic; however, it differed significantly in both context and outcome. Both audits employed a two-cycle design, implemented education-focused interventions, and evaluated key metrics, including timely imaging, diagnostic yield, and documentation quality. However, a critical procedural difference between the two institutions likely contributed to the disparity in outcomes, particularly in relation to imaging delays. At our institute, the introduction of the Emergency Department Atraumatic Loin Pain Pathway in 2015 marked a significant shift from the previous model, in which CT KUB imaging was arranged solely by urology following A&E referral and hospital admission. This pathway not only streamlined the diagnostic process but also empowered A&E clinicians to initiate timely imaging, thereby eliminating bottlenecks and reducing unnecessary delays. In contrast, the NUH audit showed persistent delays in imaging, particularly among patients referred from primary care, with only 32.0% of patients imaged within 24 hours in the second cycle. Their findings indicated barriers related to interdepartmental coordination and access to imaging, especially outside the hospital setting. Furthermore, while NUH saw improvement in CT KUB positive stone detection rates (rising from 40.5 to 65.5%), they continued to encounter vague and nonspecific imaging requests, leading to inefficiencies in scan vetting. By contrast, in our institute, the second cycle demonstrated a complete elimination of CT KUB requests for nonspecific abdominal pain and full compliance with the 24-hour imaging standard (100%), suggesting that the existing infrastructure, combined with focused educational reinforcement, enabled more effective and sustainable improvements. These differences highlight the impact of institutional protocols and referral pathways on audit outcomes, and they demonstrate how system-level changes, such as streamlined referral processes, can be instrumental in delivering timely, guideline-concordant care for patients presenting with suspected renal colic. 17

This study has some limitations. First, it was conducted at a single tertiary care center, which may limit generalizability to other settings, particularly district hospitals with different staffing or imaging resources. Second, although imaging turnaround and documentation improved, the audit did not assess clinical outcomes such as time to treatment, complication rates, resolution of symptoms, or patient satisfaction. This absence of clinical outcome data limits interpretation of the intervention's full impact on patient outcomes. Finally, despite the significant improvements observed, it remains unclear how sustainable these changes will be over the long term without continued reinforcement.

Overall, this audit has demonstrated that improving the quality of clinical information included in imaging requests, particularly through more precise documentation of relevant symptoms and risk factors, can significantly improve the radiological vetting process. As seen in our second cycle, better clinical history-taking led to a more targeted use of CT KUB, fewer imaging for nonspecific presentations, and a higher diagnostic yield for urinary stones. This approach not only minimizes unnecessary radiation exposure but also supports timely and accurate diagnosis, reducing the risk of complications associated with missed or delayed cases. While further improvements are possible, especially in sustaining these outcomes over time, this audit represents a meaningful step toward optimizing the investigation of suspected renal colic and improving patient's care through evidence-based imaging practices.

Sustaining the improvements identified by this audit will require ongoing reinforcement. Continued education such as refresher training sessions, guideline reminder posters, or brief departmental lectures, along with regular reaudit cycles are needed to maintain high compliance and prevent regression. While no formal institutional sustainability plan is currently in place, we recommend regular feedback and monitoring to embed best practices over time. Importantly, this audit model can be used by other hospitals as they could adopt the same approach to improve guideline adherence and diagnostic yield. In this way, the project provides a practical template for reinforcing high standards and supports broader clinical audits and quality improvement efforts.

## Conclusion

This clinical audit has demonstrated that targeted educational interventions and greater awareness of national imaging guidelines can significantly improve the investigation and management of suspected renal colic. By reinforcing the use of CT KUB as the first-line imaging modality in accordance with NICE, RCR, and BAUS guidance and by promoting the importance of detailed clinical histories, we achieved full compliance with the 24-hour imaging target and observed improvements in both diagnostic yield and documentation quality. These changes are expected to contribute to earlier diagnosis, timely management, and fewer avoidable complications associated with delayed or inappropriate imaging.

Although there is still room for further optimization, particularly in maintaining high standards over time, our project has laid a strong foundation for continued progress. Future audit cycles will help ensure the sustainability of these improvements and facilitate the identification of emerging challenges. Regular refresher sessions for frontline staff, departmental presentations, and visible reinforcement of clinical standards through posters and access to guidelines will remain key strategies moving forward. Ongoing evaluation with further audit cycles will be essential to build on these achievements and continue improving outcomes for patients presenting with renal colic.

**Table 1:** Patient demographics and comparison of key results before and after intervention

Variable	Level	Cycle 1	Cycle 2	Total	*p* -Value
Number		**77 (50.3%)**	**76 (49.7%)**	**153**	
Age (y) a		39.6 ± 14.2	39.0 ± 13.3	39.3 ± 13.8	0.777
Overall timing (h) a b		14.7 ± 9.5	8.2 ± 5.1	11.5 ± 8.3	0.000
	Less than 24 hours	71 (92.2%)	76 (100%)	147 (96.0%)	0.007
Gender	Male	43 (55.8%)	40 (52.6%)	83 (54.2%)	0.540
	Female	33 (42.9%)	36 (47.4%)	69 (45.1%)	
	Nonbinary	1 (1.3%)	0 (0.0%)	1 (0.7%)	
Medical history (symptoms)	Hematuria	50 (64.9%)	51 (67.1%)	101 (66.0%)	0.777
	Loin pain	70 (90.9%)	75 (98.7%)	145 (94.8%)	0.031
	Nonspecific abdominal pain	7 (9.1%)	0 (0.0%)	7 (4.6%)	0.007
	Sufficient history	59 (76.6%)	68 (89.5%)	127 (83.0%)	0.034
Stone present		32 (41.6%)	37 (48.7%)	69 (45.1%)	0.376
Alternative diagnosis		8 (10.3%)	13 (17.1%)	21 (13.7%)	0.674
Imaging	Previous CT KUB	17 (22.1%)	7 (9.2%)	24 (15.7%)	0.029

Abbreviation: CT KUB, computed tomography of the kidneys, ureters, and bladder.

a Mean ± standard deviation (SD).

b Timing from presentation to reporting.
